# Effect of Long-Acting Selenium Preparation on Health and Productivity of Sheep

**DOI:** 10.3390/ani12020140

**Published:** 2022-01-07

**Authors:** Justyna Błażejak-Grabowska, Stanisław Milewski, Katarzyna Ząbek, Przemysław Sobiech, Roman Wójcik, Katarzyna Żarczyńska, Jan Miciński

**Affiliations:** 1 Department of Commodity Science and Animal Improvement, Faculty of Animal Bioengineering, University of Warmia and Mazury in Olsztyn, Oczapowskiego 5, 10-719 Olsztyn, Poland; justyna.blazejak@uwm.edu.pl; 2 Department of Sheep and Goat Breeding, Faculty of Animal Bioengineering, University of Warmia and Mazury in Olsztyn, Oczapowskiego 5, 10-719 Olsztyn, Poland; stanmil@uwm.edu.pl (S.M.); micinsk@uwm.edu.pl (J.M.); 3 Department of Internal Diseases, Faculty of Veterinary Medicine, University of Warmia and Mazury in Olsztyn, Oczapowskiego 14, 10-719 Olsztyn, Poland; psobiech@uwm.edu.pl (P.S.); katarzyna.zarczynska@uwm.edu.pl (K.Ż.); 4 Department of Microbiology and Clinical Immunology, Faculty of Veterinary Medicine, University of Warmia and Mazury in Olsztyn, Oczapowskiego 13, 10-719 Olsztyn, Poland; brandy@uwm.edu.pl

**Keywords:** barium selenate, ewes, blood indices, lambs, meat performance

## Abstract

**Simple Summary:**

Selenium (Se) plays an important role in many biological functions, especially in the immune system. We studied the effects of injectable Se supplementation on the immune status, blood parameters, body weights and average daily gain of lambs. The results of the study indicate that the Se preparation induced an increase in the values of the parameters of both non-specific and specific immunity. Lambs from the experimental group achieved higher body weights and daily gains. The present findings can contribute to a better understanding of the biological functions of Se and improve the efficacy of this mineral in livestock production.

**Abstract:**

The aim of this study was to determine the effects of long-acting selenium (Se) preparation in sheep. The experimental material comprised Skudda ewes and their lambs. The animals were divided into two equal groups: C-control, and E-experimental. Between days 70 and 80 of pregnancy, group E ewes were injected with Se preparation (Barium Selenate Injection, BVP Animal Care, Ireland) at f 1 mL/50 kg body weight. Hematological, biochemical and immunological blood parameters as well as Se levels were analyzed in ewes. The growth rate of lambs, the dimensions of the *musculus longissimus dorsi* (MLD) cross-section, and fat thickness over the loin-eye area were also determined. It was found that barium selenate stimulated the mechanisms of humoral and cellular immunity. The injection was an effective form of Se supply, which was confirmed by its increased concentration in the blood serum of lactating ewes. The offspring of the experimental ewes were characterized by a faster growth rate, and they achieved significantly higher body weight (*p* ≤ 0.05) at 100 days of age. The lambs also had significantly higher parameters of MLD (*p* ≤ 0.05) at similar carcass fat content.

## 1. Introduction

Selenium (Se) is a trace element and its major biological form is the amino acid selenocysteine, which is an integral component of the active sites of certain selenoproteins responsible for the bioactivity of Se [1]. Plants are the richest source of this microelement for ruminants [2]. Its higher concentrations can be found in plants of the families *Amaryllidaceae* (garlic, onion) and *Brassicaceae* (broccoli, cauliflower, cabbage). On the other hand, grasses are poor in Se [3]. The Se content of plants is determined by its distribution in the soil and phytoavailability [2]. It may occur in the environment in various oxidation states, including selenates (Se VI and IV), selenides, and elemental Se [4]. The bioavailability of Se in ruminants can be limited by factors such as increased dietary sulfur intake and increased concentrations of cyanogenic glycosides in certain legumes (clover, flaxseeds) [5]. Additionally, diets rich in carbohydrates, nitrates, sulfates and calcium have a negative impact on Se utilization by cattle [6]. Microorganisms colonizing the rumen decrease Se bioavailability by reducing selenite to non-absorbable elemental Se, which is afterwards excreted with feces [5].

Feed rations for ruminants are composed almost exclusively of plant-derived components, providing them with only a marginal or an insufficient dose of Se [1]. Se deficiency has a direct or an indirect negative effect on the growth, production, and health of ruminants [6]. For example, in young animals (calves, lambs), it may lead to diet-related muscular dystrophy (MD), that is, to the hyaline degeneration of muscle fibers [7]. Se also has a strong impact on the functions of the non-specific and specific immune systems. Its stimulatory effects consist in enhancing the phagocytic and bactericidal activity of micro- and macrophages, and the activity of lysozymes. Se also plays an important role in the mechanisms conditioning the reproductive function [8].

One of Se supplementation methods whose effectiveness has been confirmed in studies of cattle [9] involves the injection of long-acting preparations, e.g., barium selenate, which release Se for several months. When administered one month prior to calving, barium selenate turned out to be an effective prophylactic agent that diminished the incidence of mastitis. Annett et al. [10] demonstrated that it improved the Se status of lambs, which was manifested by increased plasma levels of Se and enhanced glutathione peroxidase (GPX) activity, while it generally had no effect on performance parameters.

The objective of this study was to determine the effects of an injection of a long-acting barium selenate preparation on the hematological, biochemical, and immunological blood parameters and Se status of Skudda ewes, and on selected meat performance traits of their lambs. The choice of this breed was dictated by its perfect adaptation to the environmental conditions of the Warmia and Mazury Region, and its high utility value [11].

## 2. Materials and Methods

### 2.1. Animals and Nutrition

The study was conducted on a herd of Skudda sheep. Thirty ewes aged 3–4 years were divided into two equal groups: C–control (15 ewes) and E–experimental (15 ewes), based on age and body weights. The groups were formed between 70 and 80 days of pregnancy. Pregnancy was diagnosed using the Mindray DP50 scanner with an abdominal probe in the 5 MHz frequency range. Ewes from group E were subcutaneously (skin fold neck) injected with a long-acting selenium preparation (Barium Selenate Injection, BVP Animal Care, Ireland, 50 mg of Se in 1 mL) at 1 mL/50 kg body weight, which corresponds to the dosage of 1 mg Se/1 kg BW of ewes. After lambing, 20 mothers (10 in group C and 10 in group E) and 24 lambs (12 in group C and 12 in group E, 8 singles and 4 twins in each group) were selected for further research. The lambs were kept with their mothers until 100 days of age. 

During pregnancy and the lactation period, all ewes were fed the same feed. The diets for pregnant ewes were composed of meadow hay–1 kg/animal/day, barley straw–0.5 kg/animal/day and CJ concentrate–0.2 kg/animal/day. The ewes’ diets were changed during the lactation period, and they consisted of meadow hay–1.5 kg/animal/day, cereal straw–0.2 kg/animal/day and CJ concentrate–0.3 kg/animal/day. The chemical composition of the diets is presented in Table 1. The composition of CJ concentrate on a fresh-matter basis was as follows: ground barley (40%), ground wheat (37.5%), ground maize (10%), soybean meal (10%), mineral premix (2%), fodder chalk (0.2%), dicalcium phosphate (0.2%) and salt fodder (0.10%).

The lambs of both groups were fed identical diets. Apart from their mother’s milk, the lambs received meadow hay and CJ concentrate. The doses of CJ concentrate and meadow hay were increased successively every 10 days, starting from 0.05 kg at 11–20 days of age, by 0.05 kg of the concentrate and by 0.1 kg of hay. The quantities of the administered feed and leftovers were monitored to determine feed intake in each group. The nutritional value of the diets, determined based on their proximate chemical composition and energy content, was expressed in INRA units: protein digested in the intestine subject to available nitrogen (PDIN) and protein digested in the intestine subject to available energy (PDIE). The data were processed using WINWAR software. The chemical composition of feed is given in Table 1.

Hematological, biochemical, and immunological blood indices were analyzed in the 3rd month of pregnancy (day 0) and on the 28th and 100th day of ewes’ lactation. The body weights of lambs at 2, 28 and 100 days of age, and daily gains in the periods of 2–28, 28–100 and 2–100 days of age were also determined. Additionally, the dimensions of the *musculus longissimus dorsi* (MLD) cross-section and fat thickness over the loin-eye area in lambs were determined by the ultrasound method at 28 and 100 days of age.

Feed samples were subjected to chemical analyses before the experiment and three times during the experimental period. Their chemical composition (dry matter, crude protein, crude fiber, crude fat, ash) was determined using the Association of Official Analytical Chemists method [12].

### 2.2. Hematological, Biochemical and Immunological Blood Analyses 

Blood samples were collected from the jugular vein before the morning feeding. The sites of blood collection were depilated and disinfected. At each collection time, blood was collected from each dam to 2 mL K3EDTA and 9 mL serum tubes (Greiner Bio One, Kremsmünster, Austria). Hematological blood parameters were automatically analyzed using a veterinary animal blood counter (Vet Animal Blood Counter 18-Horiba ABX, Kyoto, Japan) in order to determine red blood cell count (RBC), white blood cell count (WBC), hemoglobin level (HBG), hematocrit (HCT), mean corpuscular volume (MCV), mean corpuscular hemoglobin (MCH), mean corpuscular hemoglobin concentration (MCHC) and platelet count (PLT).

In order to obtain serum samples for biochemical analysis, the samples were centrifuged immediately after collection (10 min, 3000 rpm). Biochemical parameters were determined in blood serum, within 4 h after collection, using an ACCENT 200 automated chemistry analyzer (PZ Cormay S.A., Warszawa, Poland). Biochemical tests included: the levels of glucose (GLU) and total protein (TP), the concentrations of cholesterol (CHOL), triglycerides (TG), calcium (Ca), inorganic phosphorus (P_inorg_.) and magnesium (Mg), the activity of aspartate transaminase (AST), alkaline phosphatase (ALP), lactate dehydrogenase (LDH) and gamma-glutamyl transpeptidase (GGT).

The following analytical methods were used: glucose concentration was determined with the use of glucose oxidase [13], total protein—by the biuret method [14], cholesterol-by the colorimetric method with cholesterol esterase and cholesterol oxidase [15], triglycerides—by the enzymatic method with glycerophosphate oxidase [16], AST activity—by the kinetic method [17], LDH activity—by the kinetic method with Cormay reagents [18], GGT activity—by the kinetic method with l-glutamyl-3-carboxy-4-nitroanilide [19], ALP activity—by the kinetic method [20]. All determinations were performed using an ACCENT 200 automated chemistry analyzer (Cormay) and commercial Cormay diagnostic kits. Serum Se levels were determined in triplicate by graphite furnace atomic absorption spectrometry in the LABOKLIN Laboratory for Clinical Diagnostics GmbH & Co. KG (Bad Kissingen, Germany).

Immunological determinations included humoral and cellular defense mechanisms. The following humoral immunity parameters were determined: lysozyme activity, ceruloplasmin activity and gamma globulin levels. Cellular immunity mechanisms were analyzed based on specific and non-specific immunity parameters: respiratory burst activity (RBA) after stimulation with PMA (Phorbol Myristate Acetate, Sigma-Aldrich, St. Louis, MO, USA) and the potential killing activity (PKA) of mononuclear and polymorphonuclear phagocytes, the proliferative response of blood lymphocytes (T-cells and B-cells) after stimulation with the mitogens concanavalin A (ConA) and lipopolysaccharide (LPS). 

Lysozyme activity in the blood plasma was determined by the turbidimetric method described by Siwicki and Anderson [21], and ceruloplasmin activity was measured by the method proposed by Siwicki and Studnicka [22]. The serum concentrations of gamma globulins were determined by the colorimetric micromethod proposed by Siwicki and Anderson [21]. RBA after stimulation with PMA was measured by spectrophotometry (OD 620 nm), using the method modified by Chung and Secombes [23]. The PKA of mononuclear and polymorphonuclear phagocytes was determined by spectrophotometry (OD 620 nm) according to Rook et al. [24]. The proliferative responses of T-cells stimulated with concanavalin A (ConA) and B-cells stimulated with lipopolysaccharide (LPS) were determined by MTT-ConA and MTT-LPS spectrophotometry according to Mosmann [25]. All samples were tested in triplicate, and the results are presented as mean values. The final results are presented as the reactivity index (RI). Blood parameters were determined in the analytical laboratory at the Faculty of Veterinary Medicine, University of Warmia and Mazury in Olsztyn.

The dimensions of the MLD cross-section (height, width and area) and fat thickness over the loin-eye area were determined using the Mindray DP50 ultrasound scanner with a 5 MHz linear probe. The measurements were performed in live animals according to the method proposed by Junkuszew and Ringdorfer [26].

### 2.3. Statistical Analysis

The results were processed statistically by one-way analysis of variance (ANOVA) in an orthogonal design. The results regarding blood indicators and Se concentration were analyzed in a static and dynamic system. The significance of differences between groups was verified by the Student’s *t*-test and Duncan’s test. The calculations were performed using the Statistica 13.1 program [26].

The differences among treatment groups were estimated using the following model:

Yij = μ+ αi + eij,(1)

where: Yij = dependent variable; µ = overall mean; αi = fixed effect of treatment; and eij = random residual error.

Data were evaluated by time repeated measures using the following model:

Yijk = μ + α + βj + Ti*tj + eij,(2)

where: Yijk = dependent variable; µ= overall mean; α = fixed effect of treatment; βj = effect of sampling time (on day 0, 28 or 100), and Ti*tj = fixed effect of the treatment by time interaction, eij = random residual error.

## 3. Results

### 3.1. Feed Intake

Average nutrient intake in both analyzed groups was similar throughout the entire experimental period (Table 2), which indicates that identical feeding standards were successfully maintained.

### 3.2. Hematological, Biochemical and Immunological Blood Parameters in Ewes

Table 3 presents the results of hematological analyses. A significant increase (*p* ≤ 0.05) in WBC and RBC on day 100 of lactation relative to day 0 (third month of pregnancy) was observed in group E (where ewes were injected with barium selenate). No significant differences in hemoglobin concentration or hematocrit value were found between groups. The values of MCV, MCH, MCHC and PLT counts were also similar in all animals. No significant differences in the analyzed parameters were noted on days 28 and 100 of lactation, relative to day 0.

Table 4 present the results of biochemical analyses. The barium selenate preparation caused no significant changes in the values of the analyzed biochemical parameters. The blood concentrations of GLU, TP, CHOL, TG, Ca, Mg, and P were similar in ewes from groups C and E. The activities of the analyzed enzymes, AST, ALP, LDH and GGT, were also similar in ewes from both groups. No significant changes in the analyzed parameters were found during the entire experiment.

Humoral immunity parameters are presented in Table 5. The barium selenate preparation enhanced the activity of lysozyme and increased the concentration of gamma globulins. In turn, the activity of ceruloplasmin was similar in all analyses. The activity of lysozyme in experimental ewes was significantly higher on days 28 (*p* ≤ 0.05) and 100 of lactation (*p* ≤ 0.01). In the final stage of lactation, the activity of this protein was significantly higher, compared with day 0.

The concentration of gamma globulins was similar in both groups on day 0, and it increased significantly in the experimental group in the subsequent analyses. Gamma globulin concentration was significantly higher on day 100 of lactation than on day 0.

Table 5 data suggest that the Se preparation induced an increase in the values of the parameters of both non-specific immunity-RBA and the PKA of phagocytes, and specific immunity-MTT-Con A and MTT-LPS. They reached higher values in experimental ewes, both on day 28 and 100 of lactation. A highly significant difference (*p* ≤ 0.01) in RBA was noted on day 100 of lactation, and a significant increase in PKA was observed in experimental ewes on days 28 (*p* ≤ 0.01) and 100 (*p* ≤ 0.05) of lactation. An analysis of the above values in the dynamic system revealed a stronger body response to the PKA of phagocytes. Considering both parameters of specific cellular immunity, MTT-ConA and MTT-LPS, differences were observed on days 28 and 100 of lactation relative to day 0 (*p* ≤ 0.01 and *p* ≤ 0.05, respectively). An analysis of changes over time demonstrated a stronger proliferative response of lymphocytes stimulated with MTT-ConA than MTT-LPS.

### 3.3. Blood Selenium Concentration in Ewes

**Figure 1:**
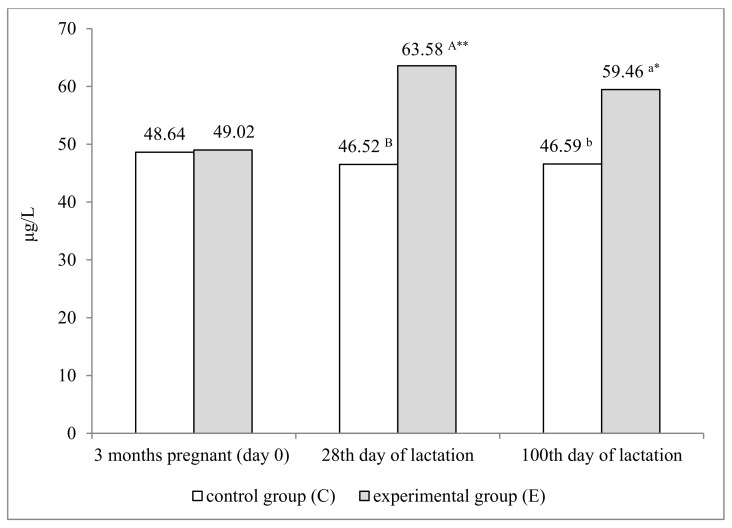
Serum selenium concentration in dams (μg/L). ^a,b^—*p* ≤ 0.05; ^A,B^—*p* ≤ 0.01; **—*p* ≤ 0.01; *—*p* ≤ 0.05—significant difference relative to day 0 (3 months pregnant).

The injection of the Se preparation led to a significant increase in serum Se concentration in ewes, which was confirmed on both analytical dates during lactation (Figure 1). At the peak of lactation (day 28), serum Se concentration reached 63.58 μg/L in group E, and it was 17.06 μg/L higher than in the control group (*p* ≤ 0.01), whereas on day 100 of lactation, it was 12.87 μg/L higher (*p* ≤ 0.05). An analysis of changes in Se concentration over time revealed that it was significantly higher in group E on both analytical dates, compared with day 0 (*p* ≤ 0.01 and *p* ≤ 0.05, respectively).

### 3.4. The Growth Rate and Ultrasound Measurements of Lambs

The growth performance of lambs is presented in Table 6. Lambs from the experimental group (E) reached higher body weights than control group (C) animals, at both 28 (*p* ≤ 0.01) and 100 (*p* ≤ 0.05) days of age. These changes resulted from significant differences in their daily gains. Group E lambs had higher daily gains in all analyzed periods, however the differences relative to the control group were significant only at 2–28 days (*p* ≤ 0.01) and 2–100 days (*p* ≤ 0.05) of age. Significant differences were also noted in muscle tissue development in lambs. Group E lambs were characterized by better developed MLD muscles (*p* ≤ 0.05) at 100 days of age. In contrast, the groups of lambs did not differ significantly in fat thickness over the loin-eye area, which is indicative of their similar carcass fat content.

## 4. Discussion

### 4.1. Hematological, Biochemical and Immunological Blood Parameters in Ewes

In the present study, a tendency to increased values of WBC and RBC was noted in ewes injected with the Se preparation. Similar results were reported by Shi et al. [27], where an oral Se supplementation at a dose above 2.0 mg Se/kg DM led to a significant increase in WBC compared with the control group. In turn, Pisek et al. [28] found that the supplementation with organic and inorganic forms of Se decreased WBC in pregnant sheep, but the noted decrease was significant only in the group receiving organic Se.

In the current study, the barium selenate preparation did not affect the blood concentrations of glucose, cholesterol, triglycerides or total protein. Similar results were obtained by Soliman et al. [29] who reported a comparable glucose level in all studied groups. The results of a study of Pomeranian revealed that barium selenate increased the blood concentrations of protein and triglycerides [30]. In turn, Abdel-Raheem et al. [31] found that sheep supplemented with Se and vitamin E had higher blood levels of glucose, total protein, and total cholesterol. El-Shahat and Abdel Monem [32], and Soliman et al. [29] observed an increase in total protein levels in sheep fed diets supplemented with Se, alone and in combination with vitamin E, which was not confirmed by Ziaei [33]. On the other hand, Saba et al. [34] demonstrated that differences in total protein concentration depended not only on Se form but also on sheep breed. In the cited study, conducted on Farafra sheep, total protein concentration was significantly higher in the group administered organic Se than in the control group, while such a dependency was not observed in the Saidi breed where protein concentration was slightly higher in groups receiving both forms of Se. Studies of goats [33] have shown that Se supplementation has no effect on the lipoprotein profile or plasma cholesterol levels. In the present study, barium selenate had no significant influence on the serum concentration of Ca, P_inorg_. or Mg. Similar results were reported by Novoselec et al. [35].

The activities of AST and GGT were similar in all groups during the experiment, and remained within the reference ranges for lambs [36]. It should be noted that AST and GGT have the highest affinity for liver tissue; therefore, the absence of significant differences in their activities between groups points to the absence of pathological changes in the liver. The activity of LDH also remained fairly stable during the experiment. This parameter should be analyzed in sheep which are prone to Se deficiency and, consequently, nutritional MD [36]. Based on the results of this study, pathological changes in muscle tissues were ruled out in all animals. A similar tendency was observed in ALP activity during the study, and the activity of this enzyme remained within the physiological range for sheep [37].

This study demonstrated that the long-acting Se preparation increased serum Se concentration in ewes, which corroborates the findings of Muñoz et al. [38]. 

The results noted in the control group indicate that these animals were deficient in Se and the obtained results ranged from 0.60 to 1.61 μmol/l. Serum Se levels of 25–50 μg/L are considered insufficient, while the optimal value falls between 120 and 150 μg/L [39]. Low concentrations of this microelement were also noted in sheep by Humann-Ziehank et al. [40] and Milewski et al. [30], who reported an average serum Se level of 45.4 and 48.70 µg/L, respectively. Much lower results were obtained by Pilarczyk et al. [41] and Karimi-Poor et al. [42], 13 µg/L and 28 µg/L, respectively. The current study revealed an improvement in the Se status of ewes administered barium selenate in the 3rd month of pregnancy, which is consistent with the results obtained by Milewski et al. [30] in a study of Pomeranian sheep.

In the present experiment, barium selenate had a significant influence on the defense mechanisms of ewes, which was manifested by an increase in the activity of lysozyme and ceruloplasmin, as well as in the serum concentrations of gamma globulins and the indicators of cellular immunity: RBA, PKA, MTT-Con A and MTT-LPS. These results clearly point to an increase in the immune system’s readiness in sheep supplemented with the Se preparation. In a study of Pomeranian sheep, Milewski et al. [30] also noted the enhancement of the immune response after a single injection of barium selenate to pregnant ewes. The results obtained by Hamam and Abou-Zeina [43] suggest that the parenteral supplementation with both Se and vitamin E can significantly increase the blood concentrations of natural antioxidants in sheep. The stimulatory effect of the Se preparation on the defense mechanisms, observed in the present study, confirms the findings of other authors [1] who noted enhanced phagocytic activity in sheep regardless of the form of supplemental Se (organic or inorganic).

### 4.2. The Growth Rate and Ultrasound Measurements of Lambs

The present study revealed a beneficial influence of barium selenate supplementation in ewes on the meat performance of their offspring. Experimental group lambs were characterized by a faster growth rate and better muscle development. The same effects were noted in an earlier study by Milewski et al. [30]. Moreover, similar results were obtained by Muñoz et al. [38] who found that an injection of barium selenate administered to ewes enhanced the growth rate of their lambs. The results of the present study are consistent with the findings of El-Shahat and Abdel Monem [32], and Soliman et al. [29] who demonstrated that injections of Se and vitamin E administered to ewes significantly increased the daily gains and body weights of their lambs at weaning. The positive influence of Se supplementation on the growth rate of lambs can be attributed to its antioxidant effect because Se as selenocysteine is a component of GPX, which provides protection against peroxides [32]. The most important effects exerted by Se include an increase in GPX activity and prevention against oxidative stress in animals.

The results of this study indicate that the lambs born to the ewes that had been injected with a long-acting Se preparation were characterized by a faster growth rate and better development of MLD, which points to their higher meatiness. The observed changes were not accompanied by increased carcass fatness, which is an important consideration from the consumer’s point of view.

## 5. Conclusions

In conclusion, it was found that an injection of barium selenate administered to ewes in the third month of pregnancy seems to be an effective form of Se supply over a longer period of time, which was confirmed by increased serum Se concentration during 100 days of lactation. Barium selenate enhanced the humoral and cellular immune responses of ewes. The lambs born to the mothers supplemented with Se were characterized by a faster growth rate and better muscle development, without significant changes in carcass fatness.

## Figures and Tables

**Table 1:** Chemical composition (% of fresh matter).

Specification	CJ Concentrate	Barley Straw	Meadow Hay
Dry matter	88.65	95.21	84.95
Crude ash	5.59	4.81	9.79
Crude protein	19.32	4.22	17.25
Crude fat	3.42	1.84	1.82
Crude fiber	6.92	45.4	28.62
Gross energy MJ kg^−1^	16.14	16.89	16.22

**Table 2:** Average daily nutrient intake in groups (g/day).

Specification	Group		SEM	*p*-Value
	C	E		
Ewes				
Pregnancy: Dry matter	1187.14	1200.33	1.064	0.376
Crude protein	100.34	99.24	0.824	0.213
Crude fiber	395.64	400.03	0.664	0.145
PDIN	61.91	62.60	0.654	0.433
PDIE	79.83	79.83	0.654	0.934
Lactation: Dry matter	1534.25	1551.30	1.064	0.385
Crude protein	149.72	148.08	2.478	0.425
Crude fiber	462.88	468.02	0.868	0.356
PDIN	92.29	93.32	1.256	0.215
PDIE	112.76	112.76	0.224	0.995
Lambs				
Rearing period: Dry matter	548.63	554.73	4.856	0.426
Crude protein	70.46	69.69	0.551	0.174
Crude fiber	108.03	109.23	1.580	0.572
PDIN	43.46	43.94	0.270	0.562
PDIE	49.69	49.69	0.456	0.941

PDIN-protein digestible in the small intestine when rumen fermentable nitrogen is limiting, PDIE-protein digestible in the small intestine when rumen fermentable energy is limiting.

**Table 3:** Hematological parameters.

Parameter	3rd Month of Pregnancy (Day 0)		28th Day of Lactation		100th Day of Lactation		SEM	*p*-Value		
	C	E	C	E	C	E		Treatment	Time	Treatment × Time
WBC (10^9^/L)	7.29	7.32	7.34	7.58	7.38	7.84 *	1.086	0.036	0.749	0.789
RBC (10^12^/L)	11.42	11.45	11.46	11.59	11.43	11.65 *	0.571	0.044	0.509	0.722
HGB (g/dL)	11.75	11.83	11.86	11.92	11.83	11.96	0.346	0.513	0.931	0.983
HCT (%)	33.75	34.03	33.94	34.28	34.18	34.45	3.840	0.916	0.425	0.917
MCV (fl)	32.04	31.12	30.76	30.33	31.03	30.81	0.360	0.830	0.343	0.965
MCH (pg)	10.21	10.34	10.24	10.29	10.29	10.30	0.142	0.609	0.691	0.881
MCHC (g/dL)	34.10	34.12	34.33	33.98	34.28	34.39	0.500	0.971	0.838	0.963
PLT (10^9^/L)	512.62	509.38	516.22	518.47	519.03	523.87	19.28	0.876	0.803	0.961

*—*p* ≤ 0.05 relative to day 0, SEM—standard error of measurement. WBC—white blood cell count, RBC—red blood cell count, HBG—hemoglobin, HCT—hematocrit, MCV—mean corpuscular volume, MCH—mean corpuscular hemoglobin, MCHC—mean corpuscular hemoglobin concentration, PLT—platelet count.

**Table 4:** Biochemical parameters.

Parameter	3rd Month of Pregnancy (Day 0)		28th Day Of Lactation		100th Day of Lactation		SEM	*p*-Value		
	C	E	C	E	C	E		Treatment	Time	Treatment × Time
GLU (mmol/L)	2.23	2.27	2.26	2.24	2.24	2.28	0.126	0.251	0.936	0.396
TP (g/L)	66.28	66.39	67.02	68.51	66.98	67.66	8.45	0.474	0.962	0.203
Chol (mmol/L)	2.15	2.16	2.06	2.18	2.13	2.14	0.053	0.204	0.439	0.886
TG (mmol/L)	0.56	0.58	0.52	0.59	0.63	0.66	0.006	0.158	0.690	0.568
Ca (mmol/L)	2.31	2.30	2.33	2.34	2.30	2.29	0.019	0.223	0.816	0.482
Mg (mmol/Ll)	1.06	1.09	1.04	1.12	1.06	1.07	0.008	0.560	0.735	0.512
P_inorg_ (mmol/L)	2.64	2.63	2.78	2.59	2.64	2.79	0.472	0.281	0.770	0.198
AST (U/L)	125.80	122.43	128.70	123.98	125.55	120.27	21.39	0.275	0.879	0.169
ALP (U/L)	151.15	152.66	152.98	153.53	151.67	152.77	20.92	0.645	0.581	0.824
LDH (U/L)	1332.60	1228.04	1354.55	1245.56	1355.20	1226.98	74.54	0.553	0.187	0.690
GGT (U/L)	53.95	53.87	54.02	52.50	52.77	54.17	9.57	0.582	0.864	0.518

SEM—standard error of measurement. GLU—glucose, TP—total protein, Chol— cholesterol, TG—triglycerides, AST—aspartate transaminase, ALP—alkaline phosphatase, LDH—lactate dehydrogenase, GGT—gamma-glutamyl transpeptidase.

**Table 5:** Immunity parameters.

Parameter	3rd Month of Pregnancy (Day 0)		28th Day of Lactation		100th Day of Lactation		SEM	*p*-Value		
	C	E	C	E	C	E		Treatment	Time	Treatment × Time
Lysozyme activity (mg/L)	1.09	1.08	1.08 ^b^	1.13 ^a^	1.12 ^B^	1.32 ^A,^**	0.101	0.001	0.005	0.360
Ceruloplasmin activity (mg/L)	49.58	49.33	50.02	51.22	50.67	52.35	8.27	0.125	0.126	0.428
Gamma globulin level (g/L)	28.93	29.02	29.60 ^b^	31.04 ^a^	29.89 ^B^	33.34 ^A,^**	9.48	0.005	0.001	0.836
RBA (OD 620 nm)	0.48	0.49	0.50	0.53	0.49 ^B^	0.56 ^A^	0.051	0.001	0.273	0.939
PKA (OD 620 nm)	0.40	0.39	0.39 ^B^	0.45 ^A,^*	0.40 ^b^	0.44 ^a,^*	0.048	0.001	0.048	0.207
MTT-Con A (RI)	1.20	1.22	1.23 ^B^	1.36 ^A,^*	1.24 ^B^	1.36 ^A,^*	0.069	0.001	0.029	0.336
MTT-LPS (RI)	1.09	1.08	1.02 ^b^	1.10 ^a,^**	1.04 ^b^	1.12 ^a,^**	0.041	0.038	0.001	0.936

^a,b^—*p* ≤ 0.05; ^A,B^—*p* ≤ 0.01; *—*p* ≤ 0.05 relative to day 0; **—*p* ≤ 0.01 relative to day 0. RBA—respiratory burst activity, PKA—potential killing activity, MTT-ConA—proliferative response of T-cells stimulated by mitogen concavalin A, MTT-LPS -proliferative response of B-cells stimulated by mitogen lipopolysaccharide.

**Table 6:** Growth rate and ultrasound measurements of lambs.

Specification	Group		SEM	*p*-Value
	C	E		
Body weight (kg), days of age				
2	2.09	2.07	0.09	0.89
28	9.53 ^B^	11.17 ^A^	0.33	0.01
100	13.79 ^b^	16.09 ^a^	0.52	0.02
Daily gains (g), days of age				
2–28	286.15 ^A^	350.00 ^B^	12.65	0.01
28–100	59.17	68.33	7.25	0.53
2–100	119.39 ^a^	143.06 ^b^	5.59	0.03
Ultrasound measurements				
MLD USG scanning: - depth (cm), days of age				
28	0.83	0.89	0.03	0.60
100	1.10 ^b^	1.22 ^a^	0.05	0.01
- width (cm), days of age				
28	2.09	2.13	0.07	0.20
100	2.68 ^b^	2.76 ^a^	0.09	0.02
- area (cm^2^), days of age				
28	2.82	2.89	0.15	0.26
100	4.28 ^b^	4.37 ^a^	0.24	0.04
Fat thickness over the loin-eye area (cm), days of age				
28	0.08	0.09	0.01	0.18
100	0.15	0.17	0.01	0.06

^a,b^—*p* ≤ 0.05; ^A,B^—*p* ≤ 0.01.

## Data Availability

Not applicable.
